# *Borrelia miyamotoi* infection negatively impacts pregnancy outcomes in immunodeficient mice

**DOI:** 10.3389/fimmu.2026.1870226

**Published:** 2026-07-06

**Authors:** Alexia A. Belperron, Jialing Mao, Carmen J. Booth, Linda K. Bockenstedt

**Affiliations:** 1 Department of Internal Medicine, Section of Rheumatology, Allergy and Immunology, Yale School of Medicine, New Haven, CT, United States; 2 Department of Comparative Medicine, Yale School of Medicine, New Haven, CT, United States

**Keywords:** *Borrelia miyamotoi*, *Ixodes* ticks, mouse, pregnancy, relapsing fever, spirochetes

## Abstract

**Introduction:**

Soft tick relapsing fever (RF) infections cause adverse pregnancy outcomes, but the impact of hard tick RF on pregnancy is unknown. We assessed pregnancy outcomes in a mouse model of hard tick RF using the *Borrelia miyamotoi* clinical isolate CT14D4.

**Methods:**

Litter sizes, pup survival and presence of spirochetemia were assessed in pups from immunodeficient (*Rag1-/-*) dams infected by needle inoculation or tick transmission both before and after mating, and from wild type (WT) dams infected by needle inoculation prior to breeding.

**Results:**

Outcomes of pregnancies in immunodeficient mice revealed reduced numbers of live pups delivered and/or pup survival to weaning, independent of the route of infection, in comparison with uninfected dams. With tick-transmitted infection, we assessed the number of viable fetal-placental units present at embryonic gestation day 18.5 and found higher numbers of embryonic pups than live pups delivered in a similarly infected group of dams followed to term. Spirochetes were not detected in blood or tissues from day 18.5 embryos but were found within yolk sacs and placentae. Infection in weanling mice from immunodeficient dams was rare regardless of timing or route of infection of the dams, and likely due to spirochetes transmitted during delivery, not *in utero*. Complete blood cell counts performed at various time points after infection revealed that infection led to anemia which was more severe in pregnant mice. In contrast to immunodeficient mice, the pup survival rate in WT mice infected by needle inoculation with CT14D4 prior to pregnancy was high. Low levels of *B. miyamotoi*-specific antibodies were detected in weaned pups, which may be of maternal origin.

**Discussion:**

Our results demonstrate that *Borrelia miyamotoi* infection has adverse effects on fetal viability and pup survival in immunodeficient *Rag1-/-* mice. Embryos, however, did not harbor spirochetes and the majority of pups borne from infected dams were not infected, revealing differences between hard and soft tick-RF infections outcomes during murine pregnancy.

## Introduction

Relapsing fever (RF) infections occur worldwide and are caused by several different species of *Borrelia* spirochetes. The bacteria are transmitted by members of the arthropod phylum, mainly soft and hard body ticks (Class Arachnida) and lice (Class Insecta) (1–8). Tick-borne RF infections in people range in disease severity but often present with a sudden onset of fever lasting 1–3 days, followed by an afebrile period and then recurrent intermittent fevers. Other signs and symptoms include generalized illness, neurologic, cardiopulmonary and hematologic complications (9–11). Soft tick-borne and louse-borne RF infections can also cause severe adverse outcomes from pregnancy that have been documented with RF species found in Africa as well as those found in North America (12–19). Effects range from low birth weight and preterm births to pregnancy loss and neonatal death (1, 12, 13, 17, 19–22). Newborns can also become infected shortly after birth and are at increased risk for complications (23).

*B. miyamotoi* is an emerging RF spirochete found in hard body *Ixodes* spp. ticks that are vectors for other human diseases, including Lyme disease, babesiosis, anaplasmosis, Powassan virus, and tick-borne encephalitis (24–28). In 2011, the first human cases of symptomatic *B. miyamotoi* infection were identified in Russia and have now been reported in all *Ixodes* tick ranges worldwide (29–36). *B. miyamotoi* infection can be mild, presenting as a viral-like syndrome with fever, myalgias and arthralgias, or with more severe disease, including meningoencephalitis, in the setting of systemic immunosuppression. Little is known about the impact of *B. miyamotoi* infection on pregnancy in humans, but case reports demonstrate that infection during pregnancy can pose risks to the mother and potentially the fetus (37, 38).

We have recently established a mouse model of *B. miyamotoi* infection and disease using the clinical isolate CT14D4 obtained from the blood of a person presenting with disseminated Lyme disease (39). CT14D4 can infect WT and immunodeficient mice, including *Rag1^-/-^* mice, and infection can be acquired from and transmitted by infected larval and nymphal ticks to new mice. WT mice exhibit relapsing episodes of spirochetemia whereas mice deficient in adaptive immunity (*Rag1^-/-^* mice) develop persistent bacteremia. Here we examined the impact of CT14D4 infection on pregnancy outcomes in mice infected prior to or after conception in *Rag1^-/-^*mice. We found that *B. miyamotoi* has a negative impact on pregnancy outcomes in *Rag1^-/-^* mice independent of the route of infection - needle inoculation of infected mouse plasma or tick-transmission. Sporadic infection of offspring of *Rag1^-/-^* mice occurred, whereas no spirochetes were observed in pups from WT mice infected prior to conception. Low levels of *B. miyamotoi*-specific antibodies were detected in offspring after weaning and are likely of maternal origin.

## Materials and methods

### Mice

The following mice were purchased from Jackson Laboratories: C.129S7(B6)-*Rag1^tm1Mom^*/J (BALB/c *Rag1^-/-^)*, stock # 003145 and *BALB/cByJ* wild type (WT), stock # 00065 (40). Mice were bred and housed in filter frame cages and administered food and water *ad libitum* according to Yale University Animal Care and Use Guidelines. All mice were euthanized by carbon dioxide (CO_2_) asphyxiation using a compressed CO_2_ cylinder with flow regulator set to achieve a displacement of 30-70% of the cage volume/min, according to the American Veterinary Medical Association Guidelines for the euthanasia of animals, 2020 edition (https://www.avma.org/resources-tools/avma-policies/avma-guidelines-euthanasia-animals). The Yale University Institutional Animal Care and Use Committee approved all procedures.

### Mouse breeding

Breeding pairs consisting of up to 3 females were cohoused with a single male mouse. Female mice were assessed for vaginal plugs as a marker of successful mating and moved to single housing once plugs were observed. In some experiments the mice were cohoused during the 21-day gestation period, with the male removed only after delivery of pups. Mice used for breeding were 8–18 weeks old (https://www.jax.org/jax-mice-and-services/customer-support/technical-support/breeding-and-husbandry-support/general-husbandry-tips). All breeding mice were provided with cotton nesting material obtained from the Yale Animal Resource Center (YARC). All pups were weaned 21 days after birth and no analyses were conducted on dams or pups during the postpartum period from pup birth until the wean date.

### Infection of mice

Mice were infected via needle inoculation with infected mouse plasma (10^4^ spirochetes/mouse) or by infestation with *B. miyamotoi* infected nymphs. For needle inoculations, spirochetes in plasma aliquots from infected *Rag1-/-* mice were enumerated and the concentration adjusted with PBS to 10^4^ spirochetes/100 μl for injection subcutaneously into the lower back. For tick transmission, infected nymphs were placed in the ear canals of anesthetized mice (4 nymphs/mouse), and allowed to feed to repletion as described (41). Blood was obtained from tail vein puncture of mice to verify infection or by cardiac puncture at the time of sacrifice. Direct fluorescence assays (DFA) using FITC anti-*Borrelia* species antibodies (originally KPL, Inc, now SeraCare) were used to detect spirochetes in the blood as described (39).

### Generation of CT14D4-infected and uninfected ticks and infection of mice

The clinical isolate CT14D4 was used in all experiments (39). *B. miyamotoi*, similar to other relapsing fever spirochetes, is vertically transmitted to eggs and infected larvae continue to maintain infection after feeding and molting to adults (39, 42). The infected nymphal ticks used in these experiments were generated by first placing infected adult female ticks together with male ticks on New Zealand white female rabbits (obtained by YARC from Charles River Laboratories at 3–4 lbs) as described (43). The rabbits were housed in the animal facility for 2–4 months prior to being used as blood meal hosts for the adult ticks. Briefly, nymphal ticks that had been generated by feeding uninfected larvae on CT14D4-infected *Rag1*-/- mice (39), were fed on uninfected *Rag1*^-/-^ mice. PCR for *B. miyamotoi* DNA in representative samples of 10–20 fed nymphs revealed an infection rate of about 70%. Thirty adult female ticks were mated as described (41) and fed on rabbits by placing ticks onto one ear of the rabbit over which a small sock was placed to contain the ticks and to prevent the rabbit from dislodging the ticks once attached. Tick attachment was confirmed 1–2 days after placement by briefly removing the sock. The sock was then reattached, and the ticks were allowed to feed to repletion and detach naturally which occurred within 7 days. Fed female ticks were individually maintained and egg clutches from each female tick hatched into larvae. Testing of 10–20 larvae from individual egg clutches for the presence of CT14D4 DNA revealed some egg clutches with no detectable CT14D4 as expected based on the predicted infection rate of the adult ticks. Infection rates of larvae from egg clutches testing positive for *B. miyamotoi* DNA were 70-90%. This high level of infection was retained when larvae were fed on uninfected mice and allowed to molt to nymphs. The infection rate of nymphs used in these experiments was 80% as determined by PCR for CT14D4 DNA from 10 sample flat nymphs obtained from the larval feeding. Uninfected larvae were obtained from uninfected adult ticks fed on rabbits. Uninfected and infected larvae were allowed to feed to repletion on uninfected *Rag1^-/-^* mice to generate nymphs as previously described (44) and were stored at 8 °C and in >90% relative humidity until experimental infestations.

### Assessment of gestation day 18.5 embryos, placentae and dams

At embryonic gestation day 18.5 (determined from the time of vaginal plug positivity), pregnant mice were submitted for analyses to the Yale Comparative Pathology Research (CPR) Core in the Department of Comparative Medicine. Mice were euthanized by CO_2_ asphyxiation followed by cervical dislocation and exsanguination by cardiac puncture. Embryos and placentae were harvested from uteri, weighed and evaluated for gross changes as previously described (45). Briefly, gross evaluation of embryos included assessment of viability and the presence or absence of gross anatomical malformations. Embryos were considered alive if their hearts were beating. Dead embryos had no heartbeat. Live embryos were placed in hypothermic conditions in CO_2_ until cessation of heartbeat according to the American Veterinary Medical Association Guidelines for the euthanasia of animals, 2020 edition guidelines. All embryos were then fixed in 10% neutral buffered formalin and secondarily decalcified in Decal^®^ Solution. Embryos were trimmed and placed in cassettes, after which they were submitted to the CPR Core Histology Service for fixation, paraffin embedding, sectioning (5µm) and staining with hematoxylin and eosin (H&E) or Warthin-Starry (WS) by established methods (46).

Immunohistochemistry was performed through the Pathology Tissue Service, Yale Department of Pathology. The slides with 5µm sections were deparaffinized and rehydrated in distilled water. Heat induced epitope retrieval was performed with citrate buffer. Once cooled the slides were placed in TBS with 0.1% Tween (TBS-Tween). The primary antibody rabbit anti-CD68 (Booster Biological PA1518) was applied at a dilution of 1:100. The slides were then rinsed in TBS-Tween and horse radish peroxidase (HRP)-conjugated immunoglobulin was applied. 3,3’-diaminobenzidine was used to visualize the bound HRP, after which the slides were washed in TBS-Tween and counterstained with hematoxylin, dehydrated, cleared and mounted with resinous mounting media. The slides were examined by light microscopy and digital image recorded using a Zeiss AxioImager A1 Axioskop microscope, AxoCam MRc55 camera and AxioVision 4.7.1 imaging software (Carl Zeiss Micro Imaging, Inc. Thornwood, NY) or Olympus BX53 Microscope, DP28 Camera with CellSans Standard imaging software (Evident Scientific USA, Waltham, MA) where the reviewer was blinded to experimental conditions. Images were generated in Adobe^®^ Photoshop^®^ (Adobe Systems Incorporated, San Jose, California). Assessment of mice, tissues and slides was conducted by an expert mouse veterinary pathologist who was blinded to the different experimental groups.

### Complete blood counts

Whole blood samples from mice obtained by retroorbital eye bleed or post-mortem cardiac puncture were collected into tubes containing 0.15 mg EDTA/0.1ml of blood. Samples from individual mice were analyzed at the experimental time points described in the *Results*. Blood samples were kept at room temperature, and 50 µl of the blood/EDTA mixture was analyzed within 1 hour of collection using a Hemavet veterinary hematology analyzer at the Yale Cooperative Center of Excellence in Hematology (https://medicine.yale.edu/labmed/ycceh/).

### PCR of *B. miyamotoi*

PCR for the *B. miyamotoi flaB* gene on blood, plasma, mouse tissue or tick DNA was performed as described (39) using the forward primer 5’ gcatcattagctggaacacaagc (bp366-389) and reverse primer 5’aactggagcggctgctggagc (bp547-567) at 66°C annealing temp.

### Immunoblot

Immunoblots to detect IgM and IgG to *B. miyamotoi* lysate proteins performed as previously described (39, 47). Briefly, sera were separated from blood samples collected from dams on the day of pup weaning and from pups 2 and 6 weeks after weaning. Sera were stored for up to 2 months at 4°C prior to performance of immunoblots. Sera samples were assessed individually at a dilution of 1:100.

### Statistical analyses

Chi-Square analyses of contingency tables were used to assess differences in number of litters with live births and pup survival to weaning from infected and uninfected dams. Comparisons of pup and placenta weights were assessed using Mann-Whitney t-tests. Statistical significance of the differences between cell counts and hemoglobin levels in uninfected and infected, pregnant and non-pregnant mice were determined with Mann-Whitney t-tests.

## Results

### Infection of immunodeficient mice by needle inoculation after conception reduces pup survival

**Figure 1:**
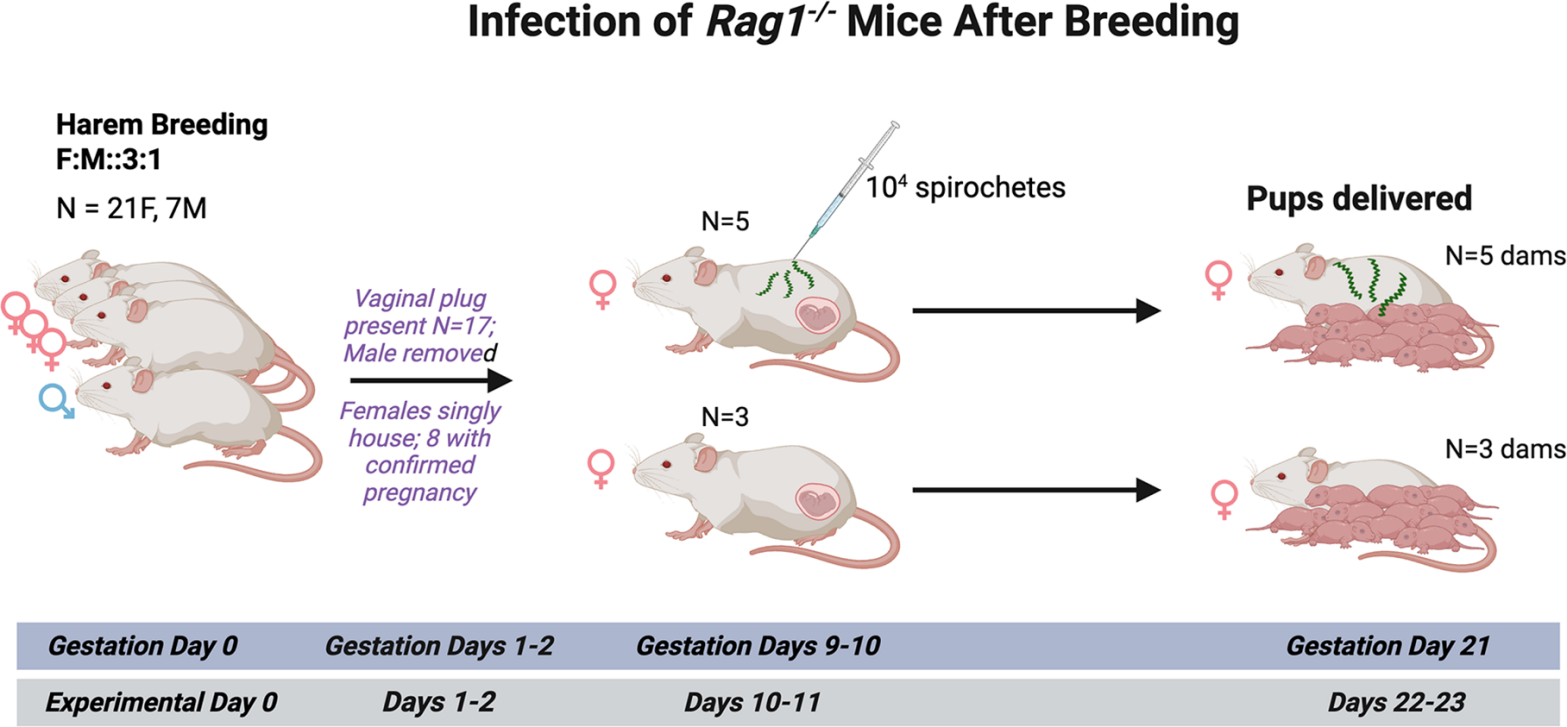
Schematic representation of experimental infection of female *Rag1*^-/-^ mice by needle inoculation of CT14D4 after pregnancy is established. Twenty-one female *Rag1*^-/-^ mice were harem bred with 7 *Rag1*^-/-^ male mice at 3:1 female-to-male ratio (7 breeding pairs). Vaginal plugs were observed in 17 of the 21 female mice within 2 days of cohousing, males were removed and females singly housed. At gestation day 9, only 8 of the 17 female mice had confirmed pregnancies. Five dams were inoculated with 10^4^ spirochetes/mouse as described in *Methods* and 3 remained uninfected. Pregnancies were followed to term (gestation day 21), at which time litter sizes were assessed for comparison with numbers of pups surviving to weaning at day 21 after birth. Blood samples were collected from pups at the time of weaning to analyze for evidence of infection. Created with Biorender.com. Bockenstedt, L. (2026) https://BioRender.com/slnli76.

Establishment of a more robust mouse model of *B. miyamotoi* infection with the clinical isolate CT14D4 is described in a companion article in this issue (39) and has facilitated more detailed studies of *B. miyamotoi* disease pathogenesis. *B. miyamotoi*-infected mice deficient in adaptive T and B cell immunity (*Rag1^-/-^* and SCID mice) remain persistently bacteremic, whereas *WT* mice clear infection within 2–3 weeks (39, 48). To determine the impact of *B. miyamotoi* infection on pregnancy outcomes, we began our studies using BALB/c *Rag1^-/-^* mice to ensure that all the dams were persistently bacteremic throughout the gestational period. This mouse strain was chosen as in our animal facility, pregnant *Rag1^-/-^* dams on this background often have larger litter sizes compared to other commonly used *Rag1^-/-^* laboratory mouse strains (Carmen Jane Booth, personal communication). Prior studies have demonstrated that BALB/c mice are susceptible to *B. miyamotoi* infection (48). Twenty-one female BALB/c *Rag1^-/-^* mice were harem bred (3 females/male), with females moved to single housing as soon as a vaginal plug was observed (within 2 days of cohousing) (**Figure 1**). Plugs were observed in 17 females but ultimately 8 were successfully impregnated. On day 9 of gestation, 5 dams were needle inoculated with 10^4^ CT14D4 spirochetes (as described in the Methods), and the other 3 dams remained uninfected. This timing was chosen based on prior published studies examining the impact of the relapsing fever *Borrelia duttonii* on pregnancy in a mouse model (49). The 3 uninfected dams produced a total of 15 live pups (litter size range 3-6) compared to 16 pups (litter size range 1-5) from the 5 infected dams, a non-significant difference. There was a significant decrease in the survival of pups to weaning, however, with 10 pups from the uninfected dams surviving and only 3 from infected dams (P = .0069, Chi-square analysis). None of the surviving 3 pups were infected at the time of weaning as assessed by DFA of blood smears (**Supplementary Table 1**; **Experiment 1**).

### Immunodeficient mice infected by needle inoculation prior to breeding have fewer live births

**Figure 2:**
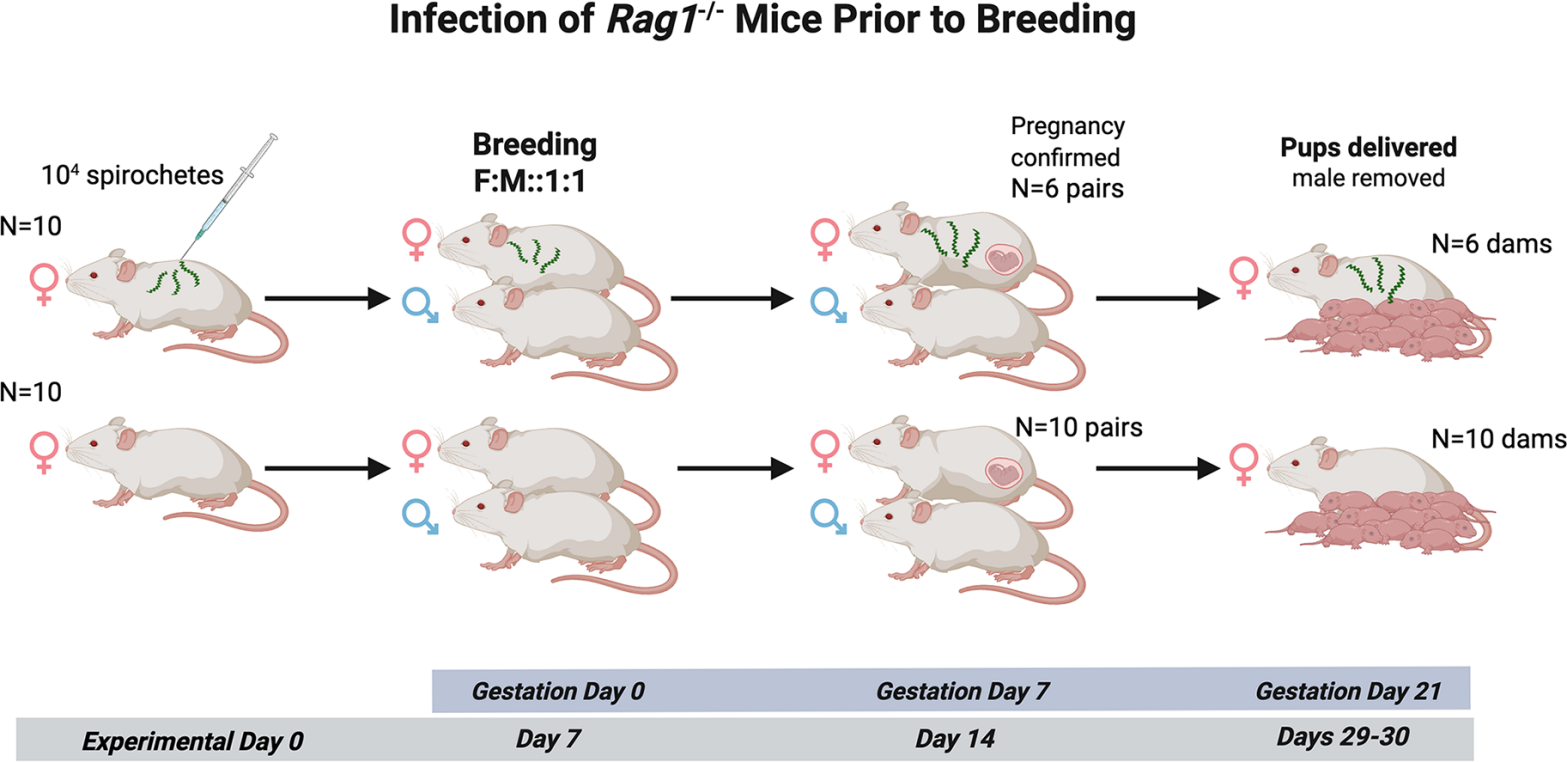
Schematic representation of experimental infection of female *Rag1*^-/-^ mice by needle inoculation of CT14D4 prior to breeding. Ten female *Rag1*^-/-^ mice were inoculated with 10^4^ spirochetes/mouse as described in *Methods* and 10 remained uninfected. Infected and uninfected female mice were bred with *Rag1*^-/-^ male mice at 1:1 female-to-male ratio (10 breeding pairs per group). By 7 days after cohousing, all female mice had confirmed pregnancies. Male mice were left with the dams throughout the gestational period. Pregnancies were followed to term (gestation day 21), at which time males were removed, Only 6 infected dams delivered live pups whereas all 10 uninfected dams delivered live pups. Litter sizes were assessed for comparison and the numbers of pups surviving to weaning at day 21 after birth were also compared. Blood samples were collected from pups at the time of weaning to analyze for evidence of infection. Created with Biorender.com. Bockenstedt, L. (2026) https://BioRender.com/slnli76.

We next infected 10 female *Rag1^-/-^* mice with 10^4^ spirochetes one week prior to breeding (**Figure 2**). A matched control group of 10 female mice remained uninfected. In this experiment, mice were bred monogamously (F:M ratio 1:1) and females were examined weekly for evidence of pregnancy. Males were left in the cages with the dams until delivery, as this is a technique reported to improve pregnancy outcomes (https://www.jax.org/jax-mice-and-services/customer-support/technical-support/breeding-and-husbandry-support/general-husbandry-tips). The males were uninfected at the time of breeding, and DFA of blood samples from males taken at the time they were separated from the females after delivery confirmed that they remained uninfected. Six of the 10 infected mice became pregnant compared to 10/10 uninfected mice bred at the same time (P = 0.025 Chi-square analysis). Although overall there were fewer live births from the infected *Rag1^-/-^* dams, the percentage of pups surviving to weaning was not significantly different compared to those from the uninfected *Rag1^-/-^* dams (24/34 compared to 52/66 respectively, P = 0.23 Chi-square analysis). Of the 24 pups that survived to weaning from the infected dams, 7 were bacteremic at the time of weaning as assessed by DFA of blood smears. The bacteremic pups were a subset of litters from 3 of the 6 dams (**Supplementary Table 1**; **Experiment 2**).

### Tick-transmitted infection of *Rag1^-/-^* mice prior to breeding results in placental but not embryo infection

**Figure 3:**
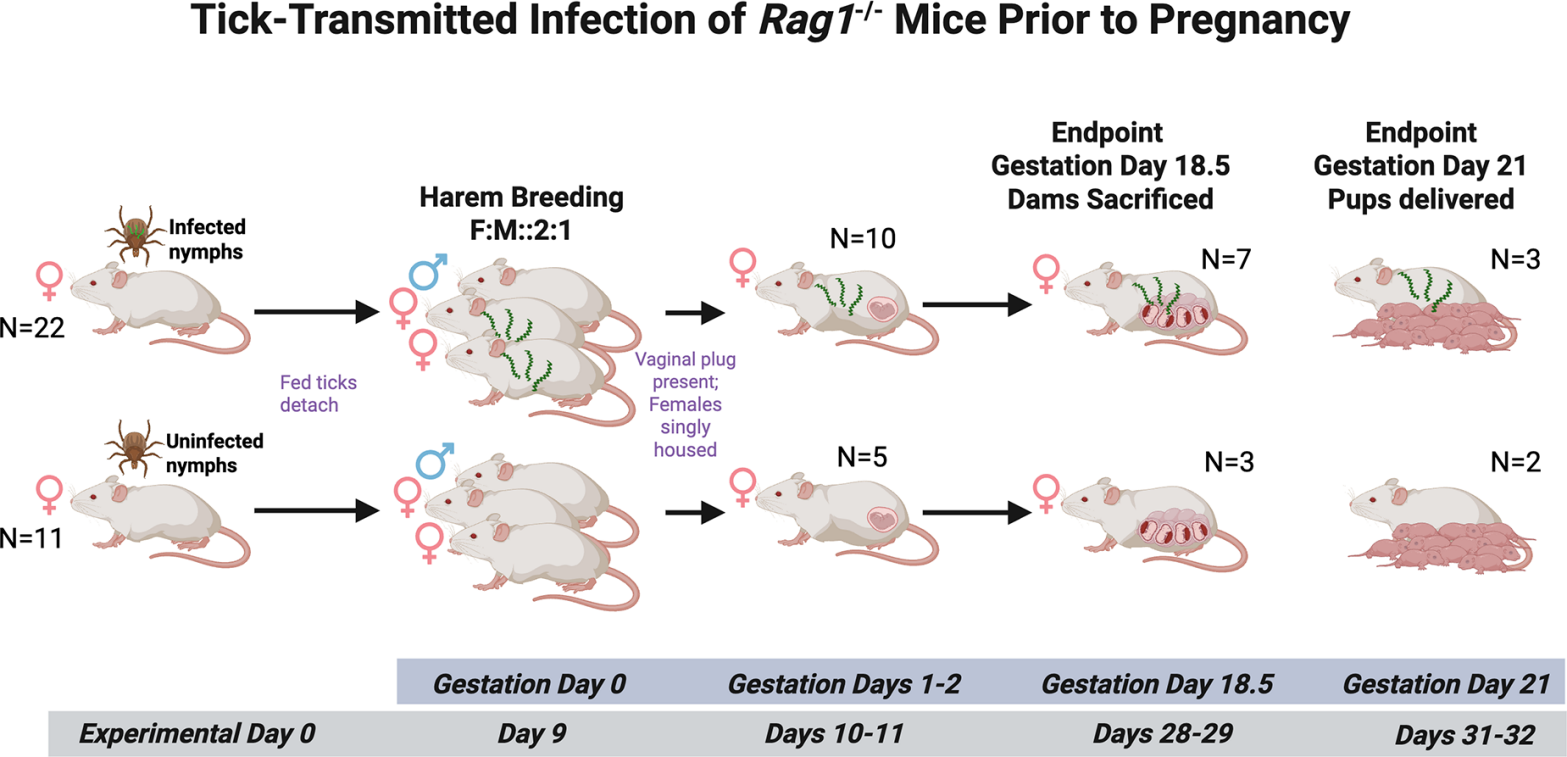
Schematic representation of tick-transmitted experimental infection of female *Rag1*^-/-^ mice prior to breeding. Twenty-two female *Rag1*-/- mice were infested with CT14D4-infected nymphal ticks (4 ticks/mouse) and 11 female mice were infested with uninfected nymphal ticks (4 ticks/mouse) as described in *Methods*. After ticks had fed to repletion and detached naturally, infected and uninfected female mice were harem bred with *Rag1*^-/-^ male mice at 2:1 female-to-male ratios. Females with vaginal plugs were singly housed, with 10 infected and 5 uninfected female mice having confirmed pregnancies. Seven of the 10 infected dams and 3 of the uninfected dams were sacrificed at gestation day 18.5 for histopathologic analysis of embryos *in utero*. The remaining 3 infected and 2 uninfected dams were followed to term (gestation day 21) and litter sizes were assessed for comparison with numbers of pups surviving to weaning at day 21 after birth. Blood samples were collected from pups at the time of weaning to analyze for evidence of infection. Created with Biorender.com Created in BioRender. Bockenstedt, L. (2026) https://BioRender.com/slnli76.

Initial experiments described above were conducted with needle inoculation of spirochetes, a common and convenient mode of laboratory infection which permits use of a defined number of spirochetes. However, it is not a natural route of infection, so the pregnancy studies were repeated with tick-transmitted infection (see experimental design, **Figure 3**). As the prior experiments revealed possible defects in fetal pup development or survival *in utero* as well as neonatal pup survival to weaning, we assessed embryos and placentae at day 18.5 of gestation as well as outcomes of pups delivered from infected or uninfected dams (**Figure 3**; **Supplementary Table 1**; **Experiment 3**).

Female *Rag1^-/-^* mice were infested with CT14D4-infected nymphs (4 ticks/mouse, N = 22 mice) or uninfected nymphs (N = 11 mice) as controls, as previously described (50) (**Figure 3**). DFA on blood smears at day 7 confirmed the presence of spirochetes in all the mice infested with CT14D4-infected ticks. Nine days after tick infestation, mice were harem bred (F:M ratio of 2:1) and the following day dams with vaginal plugs were separated from male mice to ensure timing of fertilization. Ten infected and 5 uninfected mice were observed to have vaginal plugs. Of those, 7 infected and 3 uninfected dams were sacrificed at day 18.5 of embryo development, and the remaining dams were left to deliver pups at full term.

**Figure 4:**
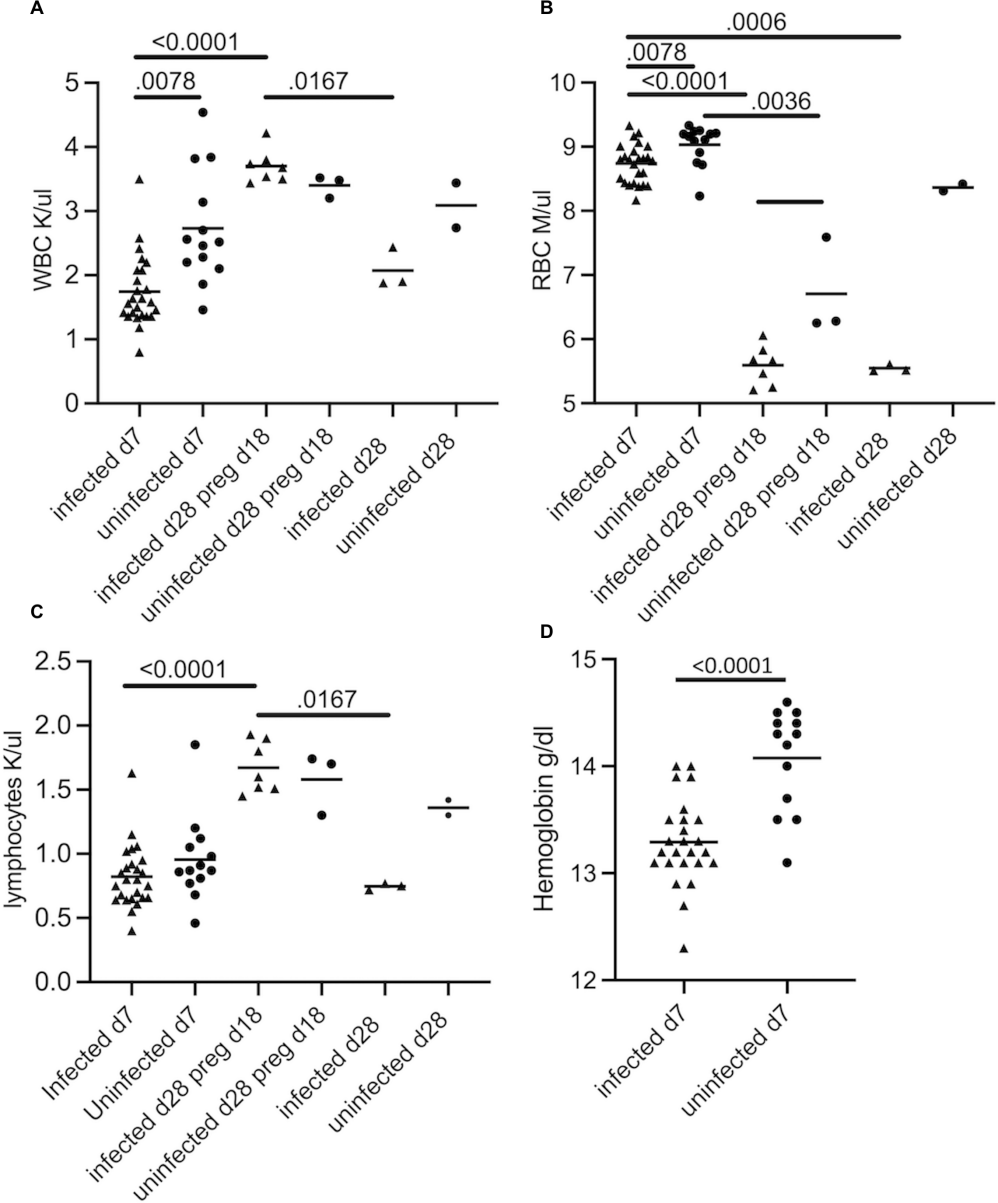
Complete Blood Cell (CBC) counts reveal differences in lymphocyte and WBC numbers and development of anemia in infected pregnant mice. CBC analyses were conducted on fresh blood samples from female mice 7 days after infestation with infected (22 mice) or uninfected (11 mice) ticks, pregnant dams 28 days after tick infestation and at gestation day 18.5 (7 dams had been infested with infected ticks and 3 dams with uninfected ticks), and nonpregnant female mice infested with infected ticks (3 mice) or uninfected ticks (2 mice) 28 days prior. All mice infested with infected ticks were confirmed to be bacteremic by day 7 after tick placement. The different female mice groups are labeled on the X axes. **(A)** White blood cells counts (WBC), K/ul= 1000 cells/microliter of blood **(B)** Lymphocyte counts **(C)** Red blood cell counts (RBC) counts, M/µl= million/microliter, and **(D)** hemoglobin levels (g/dl) in female mice at day 7 after tick-infestation were compared between mice fed on by infected ticks (22 mice-infected) or uninfected ticks (11 mice-uninfected). P values were determined by Mann-Whitney t tests and noted on the graphs with lines designating the two conditions compared.

Complete blood counts (CBC) were conducted on all mice from both the infected (N = 22) and the uninfected (N = 11) mice at day 7 after tick infestation and on 3 infected and 2 uninfected non-pregnant females 28 days after tick placement. In addition, blood was analyzed on the subgroup of dams sacrificed at day 18.5 of embryonic gestation (day 28 after tick infestation; 7 infected and 3 uninfected dams). Seven days after tick infestation, the infected *Rag1-/-* mice had reduced levels of total white blood cell (WBC) counts compared to *Rag1-/-* mice fed on by uninfected nymphs, but no differences were observed in lymphocyte numbers between the two groups (**Figures 4A, B**). Although *Rag1-/-* mice lack mature B and T lymphocytes, they maintain and can expand their natural killer and innate lymphoid cell populations (51). Pregnancy was associated with an increase in WBC and lymphocytes in both infected and uninfected dams (**Figures 4A, B**). Infection also led to anemia which was more severe in the pregnant *Rag1-/-* mice with reduction of hemoglobin and hematocrit levels (**Figures 4C, D**).

**Figure 5:**
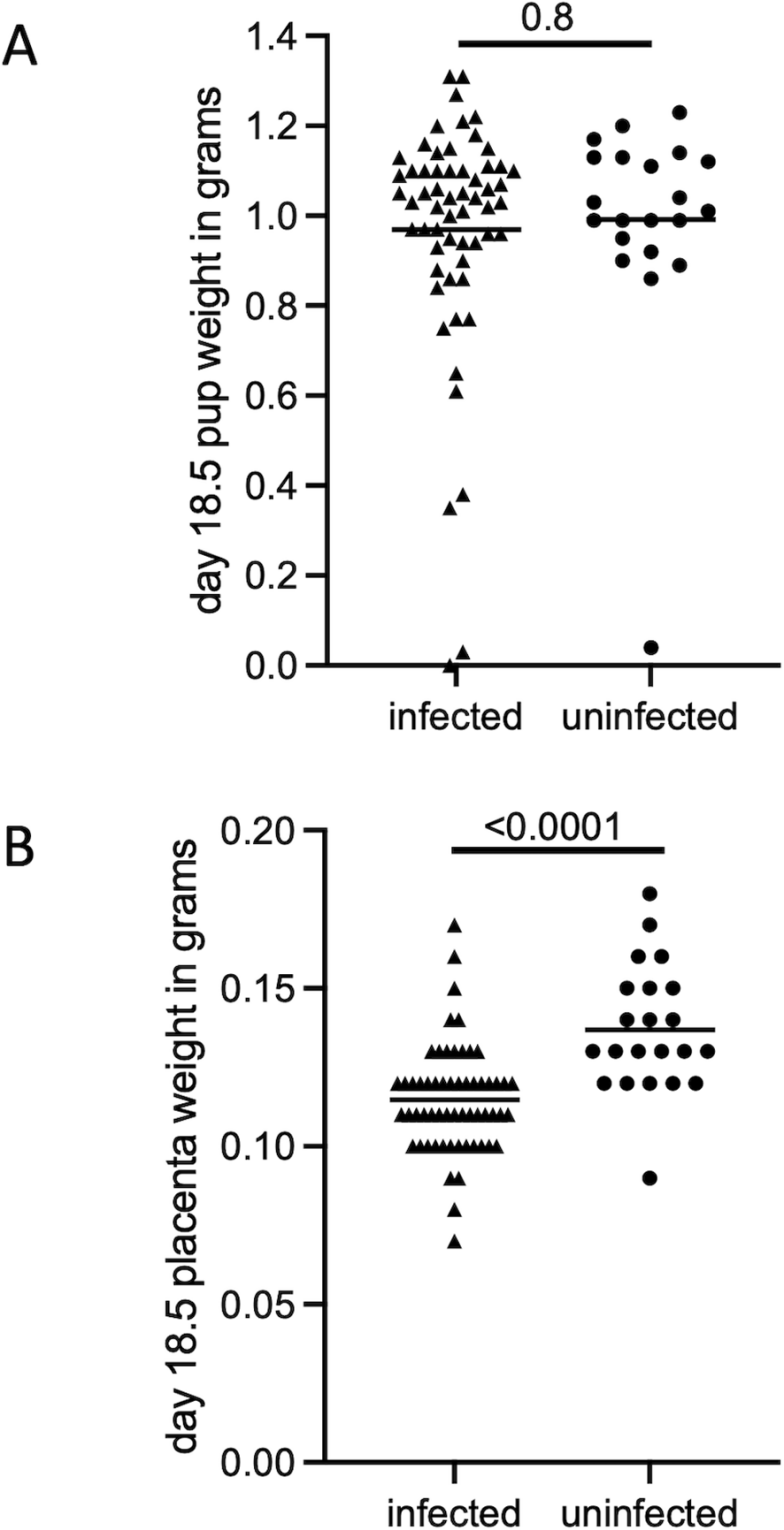
Pup and placental weights at embryonic gestation day 18.5. Seven *Rag1*^-/-^ dams infected via tick bite and 3 *Rag1*^-/-^ dams infested with uninfected ticks prior to breeding were sacrificed at gestation day 18.5 as described in *Methods* (see also **Figure 3**). Pups **(A)** and placentae **(B)** were harvested and weighed individually. P values comparing weights of the indicated tissues obtained from infected dams versus uninfected dams were determined with Mann-Whitney t tests.

H&E staining of tissue sections from pregnant dams at day 18.5 embryonic gestation were assessed by the pathologist who was blinded to the different experimental groups. Analyses revealed no significant pathologic or immunologic findings in the hearts, lungs, kidneys, spleens or brains of dams, irrespective of infection status (data not shown). Examination of the livers of 7 infected dams, however, revealed that 3 exhibited findings consistent with an inflammatory response, including, substantial increases in the numbers of megakaryocytes in the sinusoids, evidence of increased circulating cells, and increased sinusoidal congestion (52–54). Livers from 3 other infected dams had milder sinusoidal congestion, and the liver from one dam had no pathologic findings. No pathology was observed in any of the livers from the uninfected dams. Spirochetes were detected by Warthin-Starry staining of tissue sections from the hearts, lungs, and livers of all 7 infected dams, an expected finding as these mice had DFA-confirmed bacteremia and had not been perfused at the time of sacrifice (**Supplementary Figure 1**). No spirochetes were detected in tissues from the uninfected dams. Tissues from the dams served as positive and negative controls, respectively, for similar Warthin-Starry staining of embryonic pups. From the 7 infected dams, there were 61 embryonic pups (litter sizes ranged from 2–13 with a median of 10) and 22 embryonic pups from 3 uninfected dams (litter sizes ranged from 5–9 with a median of 8) (**Supplementary Table 1**, Experiment 3). Gross analysis of the placentae and of all the embryos, including the limbs, was performed as detailed previously by the pathologist (55), and did not reveal significant changes or abnormalities in the embryos from infected dams. Two embryos from each infected and uninfected dam were further assessed histologically. Two of 14 embryos from infected dams and 1 of 6 embryos from uninfected dams showed evidence of autolysis and were partially resorbed, indicating that there was little embryo death by day 18.5 gestation in either experimental group. Warthin-Starry staining was performed on four representative embryos from the infected dams and showed no evidence of spirochetes and no spirochetes were detected in 2 representative pups from the uninfected dams. There were no other significant findings or differences observed between the infected and uninfected embryos. In addition, no differences were observed in the embryo weights (**Figure 5A**); however, placenta weights from infected dams were significantly lower than those from uninfected dams (**Figure 5B**).

**Figure 6:**
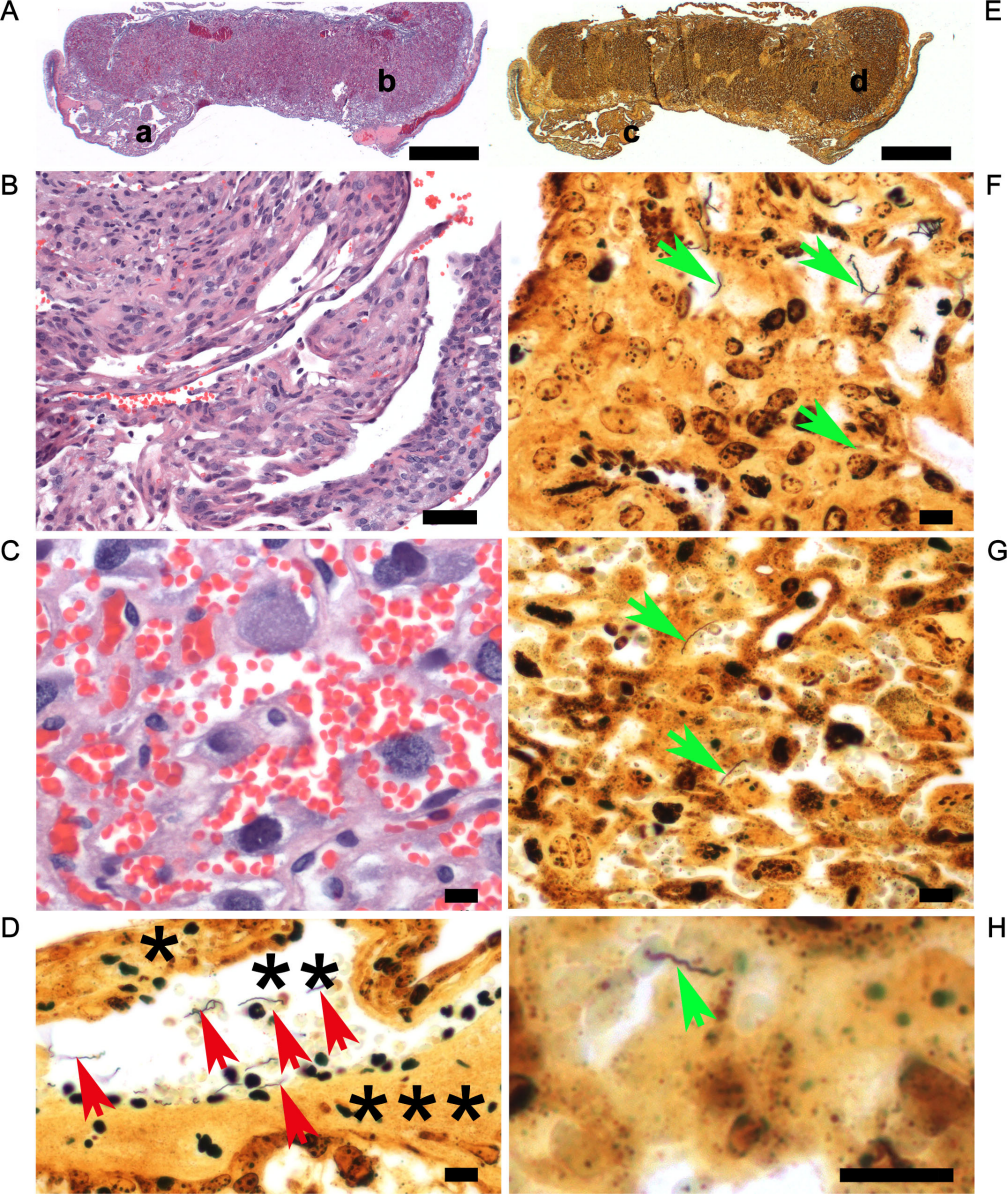
Hematoxylin Eosin (HE) and Warthin-Starry (WS) staining show a notable lack of inflammatory cells within the placenta and spirochetes within fetal tissues but not placenta respectively. Images are from stained sections of a representative placenta from a tick*-*infected *Rag1^-/-^* dam sacrificed at gestation day 18.5 as described in *Methods* (see also **Figure 3**). **(A)** Low power magnification of placenta and fetal membranes in cross section, scale bar = 1mm; **(B)** Intermediate power magnification of the fetal membranes from area a denoted in **(A)** scale bar = 200μm. **(C)** High power magnification of the placenta labyrinth from area b denoted in (A) scale bar = 10μm. Serial sections of the placenta were stained by WS for spirochetes **(D–H)**. **(E)** Low power magnification of placenta and fetal membranes in cross section, scale bar = 1mm. **(D, F–H)** use high power magnification (scale bars = 10μm). **(D)** shows numerous spirochetes within maternal blood sinus which are indicated by (**). The maternal blood sinus is located in between the uterus indicated by (*) and the placenta indicated with (***). **(F)** shows high power magnification of area c as denoted in Panel **(E)** Spirochetes (indicated with green arrows) observed within fetal membranes. **(G, H)** show high power magnification of the placenta labyrinth, area d, as denoted in **(E, H)** is zoomed in and cropped to show a single spirochete.

**Figure 7:**
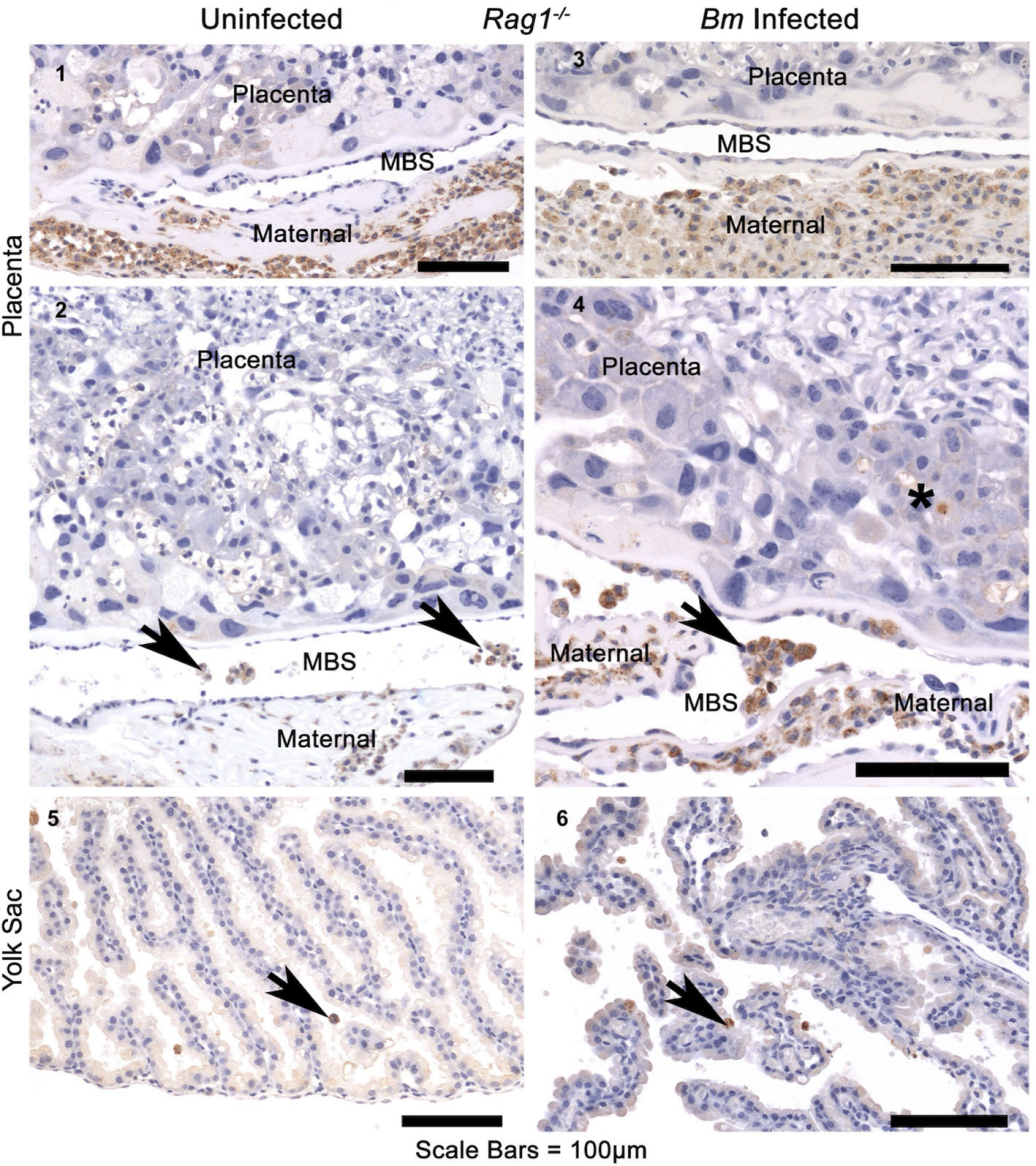
Immunohistochemistry with anti-CD68 antibodies identified small numbers of macrophages in representative sections of placentae from both uninfected and infected *Rag1^-/-^* dams. Panels 1, 3 and 5 are from dams infested with uninfected ticks, and panels 2,4, and 6 are from dams infested with infected ticks. Sections are from dams at gestation day 18.5 as described in *Methods* (see also **Figure 3**). CD68 positive cells are stained brown. There are few CD68-positive cells within the placentae and are denoted by (*). Panels 1 and 3 are placenta from uninfected dams and panels 2 and 4 are from infected dams. There are more frequent CD68-positive cells within the maternal tissues and scattered positive cells (arrows) within the maternal blood sinus (MBS) and yolk sac (panels 3 and 6) in both uninfected and *B. miyamotoi* infected mice.

Four additional tick-infected female *Rag1*-/- mice were bred (1F:1M, 3 became pregnant) as described above to provide day 18.5 embryonic gestation pups and placentae for more detailed histologic analyses (**Figure 3**; **Supplementary Table 1**; **Experiment 3**). Three embryos from each of the 3 dams (9 in total) were assessed by Warthin-Starry staining, and again no evidence of spirochetes was found even with a complete scan of the entire embryo sections. Together from the 2 separate experiments, no spirochetes were found in any of the embryonic pups examined (0/13). The 9 embryos were also analyzed histologically by H&E staining and again no evidence of abnormality or inflammation was observed. Maternal tissues were also examined by H&E staining, and no inflammatory infiltrates were found in deciduae or uteri. Spirochetes were visualized in all the maternal tissues examined by Warthin-Starry staining (**Figure 6**), including 8 of 9 placentae. To look more specifically for the possible presence of macrophage and monocytes cells (which are more difficult to define with H&E staining), placental sections were stained with anti-CD68 a marker of macrophage/monocytes. As expected, small numbers of macrophages were present, but there was no observable difference in CD68 staining levels between placenta and adjacent maternal and yoke sac tissues from infected and uninfected dams (**Figure 7**), supporting the absence of acute inflammatory infiltrates.

**Figure 8:**
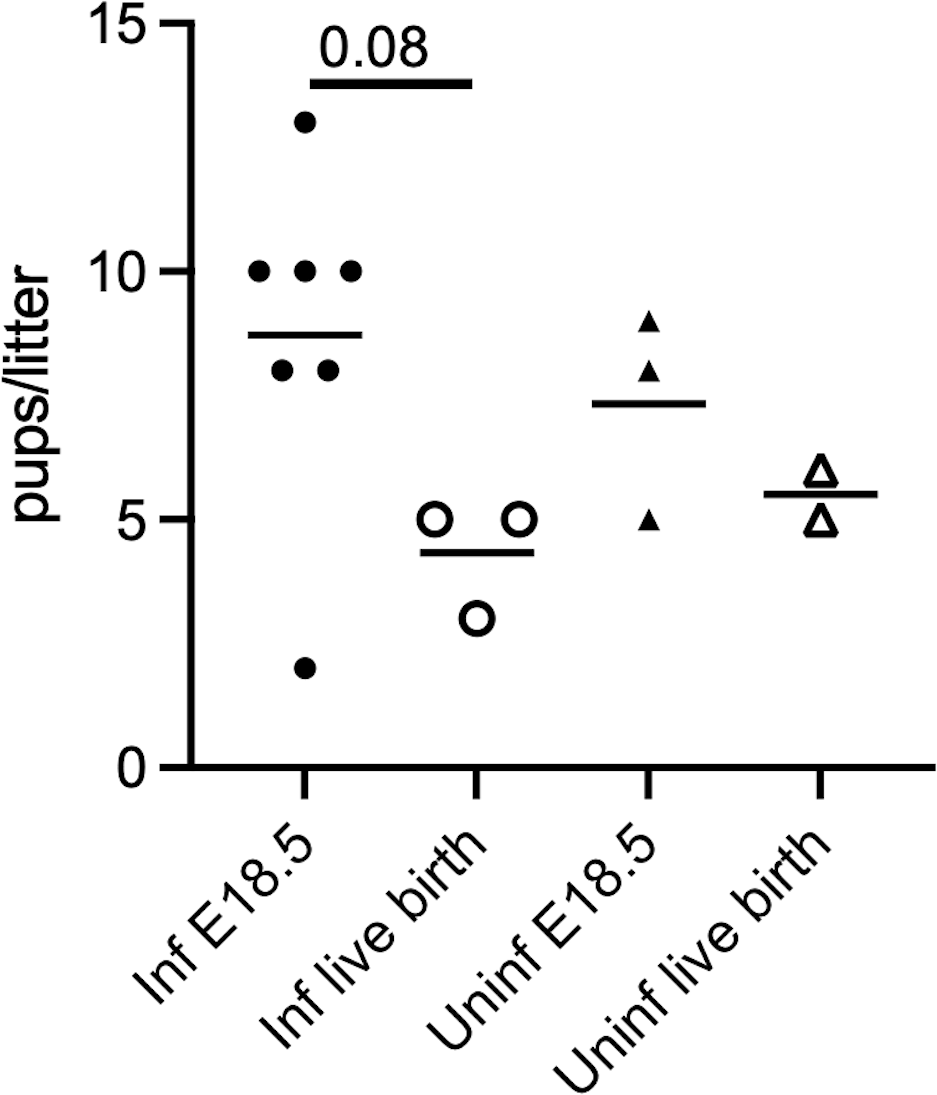
Comparison of litter sizes between embryonic gestation day 18.5 *in utero* litters from 7 infected dams (Inf E18.5) and 3 uninfected dams (Uninf E18.5) and live births from 3 infected (Inf live birth) and 2 uninfected dams (Uninf live birth) (see **Figure 3**). P value designated on graph was determined with a Mann-Whitney t-test.

Pregnancy in the remaining 3 infected and 2 uninfected dams proceeded to full term with live pup births. The mean live pup litter sizes from infected dams was 4.3 (range 3-5) which was less than the mean of the *in utero* litter size at day 18.5 of embryonic gestation (mean 8.7, range 2-13) (**Figure 8**). The means of the uninfected *in utero* and live birth litter sizes were 7.3 and 5.5 respectively (**Figure 8**). In addition, fewer of the pups from infected dams survived to weaning compared to those from uninfected dams (2/13 versus 9/11 respectively, P = .0031 Chi Square analysis). These findings were similar to those we observed with needle inoculation. One of the 2 surviving pups was bacteremic at the time of weaning (**Supplementary Table 1**; **Experiment 3**).

### Assessment of pregnancy in *B. miyamotoi* infection of WT mice

**Figure 9:**
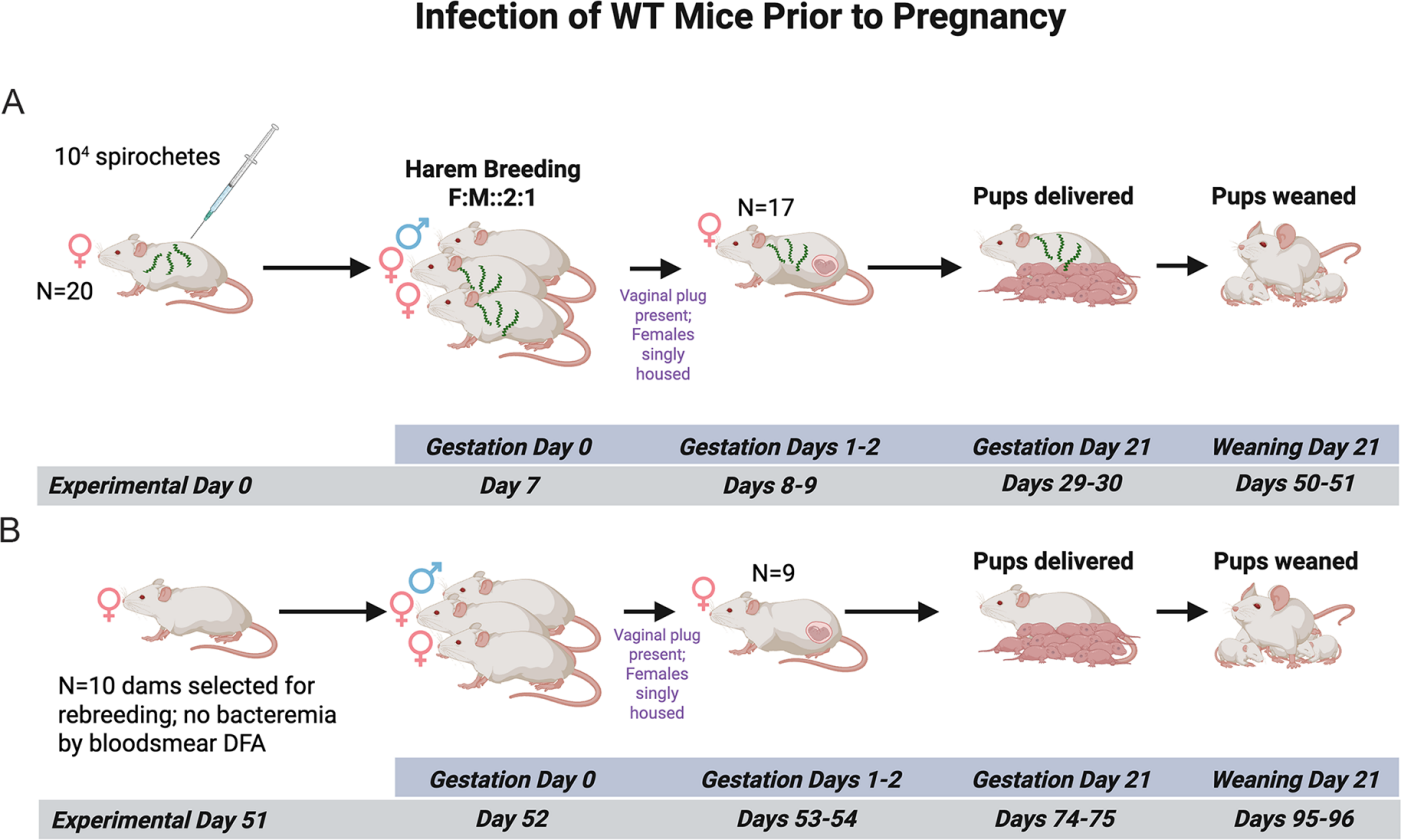
Schematic representation of experimental infection of female WT mice by needle inoculation of CT14D4 prior to breeding. **(A)** Twenty female *Rag1*^-/-^ mice were inoculated with 10^4^ spirochetes/mouse as described in *Methods*. At day 7 of infection, mice were harem bred with *Rag1*^-/-^ male mice at a 2:1 female-to-male ratio (10 breeding pairs). Females with vaginal plugs observed within 2 days of cohousing were singly housed (N = 17). Pregnancies were followed to term (gestation day 21), at which time litter sizes were assessed for comparison with numbers of pups surviving to weaning at day 21 after birth. **(B)** At the time of weaning, 11 dams were selected for rebreeding. Bacteremia had resolved in these mice by this time point (day 51 after infection). These mice were harem bred (female:male ratio of 2:1). Within 2 days of cohousing, females with vaginal plugs (N = 9) were singly housed and followed to term (gestation day 21, 75 days after initial maternal infection). Litter sizes were assessed at the time of delivery for comparison with numbers of pups surviving to weaning at 21 days after birth. Blood samples were collected from dams at the time of weaning and from pups 2 and 6 weeks after weaning. Created with Biorender.com Created in BioRender. Bockenstedt, L. (2026) https://BioRender.com/slnli76.

**Figure 10:**
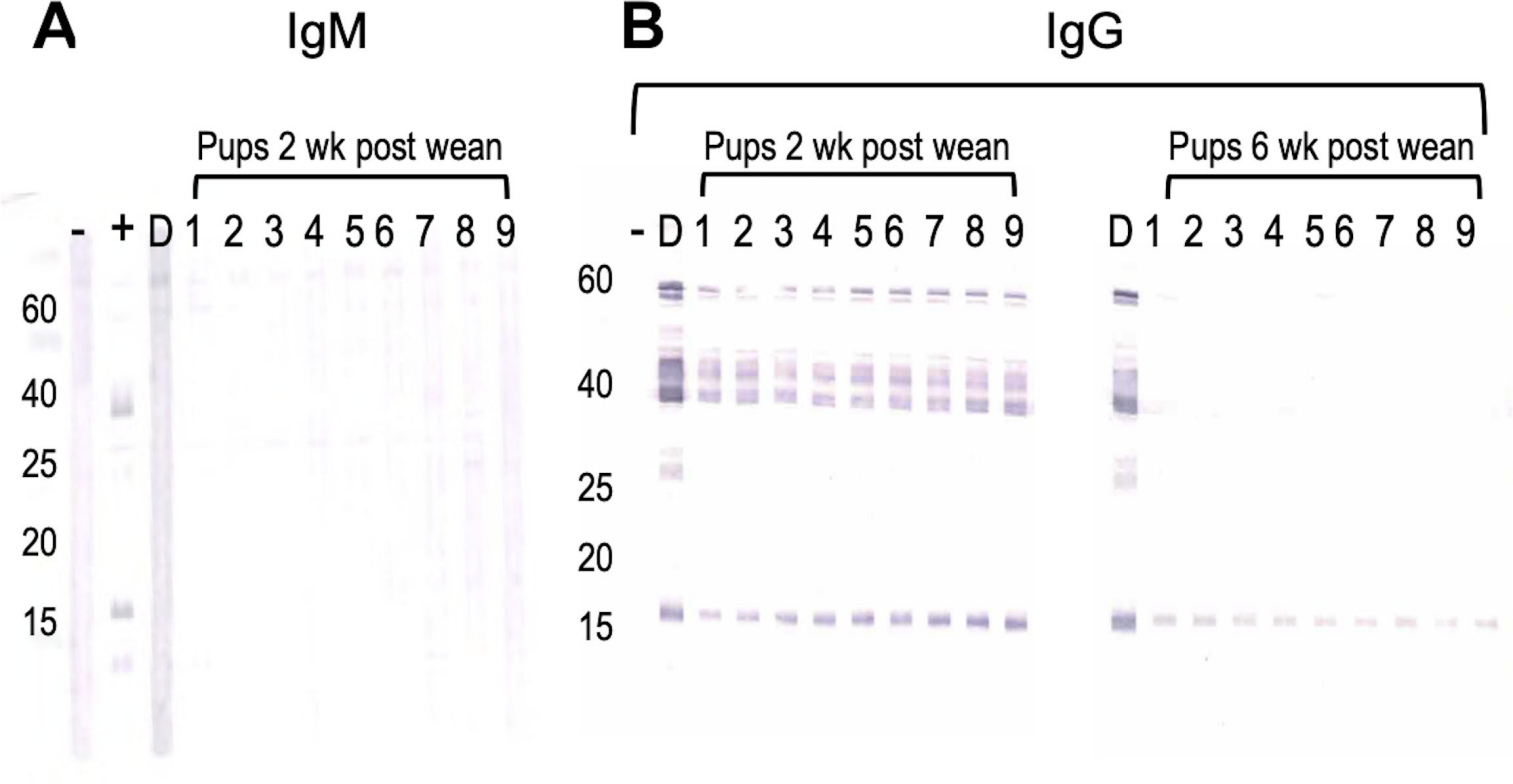
IgM and IgG immunoblots from one representative infected WT dam and weaned pups show week antibody responses in the pups that diminish overtime. The dam was bled at the time of weaning (4 weeks after infection see **Figure 9**), and the pups were bled 2 weeks and 6 weeks after weaning. **(A)** shows the IgM reactivity of the pup sera 2 weeks post wean. **(B)** shows the IgG reactivity of the pup sera at 2 and 6 weeks post wean as denoted in the Panel. In **(A)**, the “+” indicates a positive control serum sample from a 14-day infected mouse (39), and the “-” in **(A, B)** indicates a negative control serum from an uninfected mouse. “D” is the dam serum described above. Pups 1–4 are female and pups 5–9 are male. The serum from the infected dam (confirmed by blood DFA as described in methods) serves as the positive control for the IgG blots.

To assess the course of pregnancy in *B. miyamotoi* infected WT mice, 20 female mice were infected by needle inoculation with 10^4^ spirochetes and harem bred one week later (F:M ratio 2:1) (**Figure 9**; **Supplementary Table 1**; **Experiment 4**). Females were moved to single housing once a vaginal plug was observed; 17/20 mice became pregnant and all delivered live pups (range 2 – 10/litter, median 5), and 87/100 pups survived to weaning. At the time of weaning, none of the pups had detectable bacteremia by DFA of blood smears, but one tested positive by PCR for *B. miyamotoi* DNA in blood (**Supplementary Figure 2**). We also assessed IgM and IgG antibody responses by immunoblot using blood samples from dams obtained on the day their pups were weaned (4 weeks after dams were infected) and blood samples from pups obtained 2 and 6 weeks after weaning. IgM antibody responses in the dams were weak at this 4 week timepoint after infection, similar to what we have previously observed (39) so a serum sample from a 14-day infected mouse was used as a positive control. The IgM reactivity in sera of the pups 2 weeks after weaning was very low (**Figure 10A**). Sera from pups had positive IgG reactivity to multiple *B. miyamotoi* antigens, including proteins in the molecular range of vlps (~40Kd) and vsps (20Kd) (56–58) (**Figure 10B**). This reactivity was considerably weaker than maternal antibody reactivity assessed in sera collected from dams at the time of pup weaning and substantially diminished by 6 weeks (**Figure 10B**).

After the pups were weaned, 9 of the dams were successfully bred again (F:M ratio 2:1-see **Figure 9**; **Supplementary Table 1**; **Experiment 4**). By this time, 7 weeks after initial infection, all the dams had resolved bacteremia. All dams delivered live pups (range 2-11/litter, median 5), with litter sizes similar to those of the dams after their first breeding. 100% of the 51 pups born from the second breeding survived to weaning, a significantly higher rate than that from the first pregnancies (51/51 versus 87/100 respectively, P = .0071 Chi Square analysis).

## Discussion

In these studies, our goal was to examine the impact of *B. miyamotoi* infection on pregnancy outcomes in a mouse model. Studies of pregnancy in mice may share direct relevance to human disease, as humans and rodents both have discoid shaped and hemochorial placentae where maternal blood comes in direct contact with the fetal chorion, the outermost extraembryonic membrane that surrounds the developing embryo (59–61). Our establishment of a mouse model for *B. miyamotoi* infection and disease using the clinical isolate CT14D4 identified *Rag1^-/-^* mice as having persistent bacteremia and more significant pathology than WT mice, which typically resolve infection in 2–3 weeks (39). We therefore focused on examining pregnancy in CT14D4-infected *Rag1^-/-^* mice, as these mice maintain a consistently high level of bacteremia throughout the term of pregnancy which may increase the likelihood of observing adverse events. Our results documented exacerbation of maternal anemia, infection of placentae, reductions in live births and decreased neonatal pup survival to weaning. However, we only found sporadic infection in weaned pups (8 infected pups out of a total 29 weaned) from *Rag1^-/-^* dams, which showed no correlation with route or timing of dam infection. The decrease in litter sizes and pup survival led us to examine pups *in utero* to determine if embryos were infected which could contribute to their demise. Notably, we did not find evidence of spirochetes in any of the d18.5 embryos examined (0/13), even though we easily detected spirochetes in placental tissues by Warthin-Starry staining. Although mice have hemochorial placentae where maternal blood comes in direct contact with the fetal chorion, there are three layers of trophoblasts that separate maternal and fetal blood, and these cells may block spirochetes from passing into mouse embryos (60, 62). Given the absence of spirochetes by Warthin-Starry staining in embryos, we postulate that the sporadic finding of infected weaned pups is a result of contact with maternal blood during delivery. In the tick-transmitted infection experiment (**Figure 3**), we also observed a decrease in the litter sizes at full term with live births compared to the litter sizes in infected dams sacrificed at 18.5 days of gestation. Although this difference did not reach statistical significance due small total numbers of litters and variability in litter sizes, it does suggest that fetal demise may occur at the end of term beyond day 18.5 of gestation. We also did not observe any spirochetes in the blood of weaned pups from infected WT dams (0/138). These findings together with the low levels of IgG and IgM antibodies reactive with *B. miyamotoi* antigens found in the pup sera support the conclusion that pups are not infected *in utero*. The low levels of antibody detected are most likely maternal in origin, especially IgG which can cross the placenta and also be acquired by pups through maternal milk (63). The one WT weaned pup that exhibited PCR positivity for *B. miyamotoi* DNA on a blood sample had similar antibody reactivity to pups that tested negative. We speculate that if pups are exposed to *B. miyamotoi* during birth, maternally transferred antibodies may be sufficient to prevent infection establishment.

Prior studies with the CT14D4 clinical isolate of *B. miyamotoi* (39) revealed that tick transmission resulted in pathology in multiple organs that was most severe in the livers after infection of C57BL/6J *Rag1^-/-^* mice. The studies reported here were performed in BALB/c *Rag1-/-* mice, and pathology was assessed 4 weeks after tick transmission of infection. We found evidence of inflammation in the liver with increased circulating cells including megakaryocytes within the sinusoids and sinusoidal congestion in 6 of 7 infected dams, but not the severe pathology observed in more chronically infected C57BL/6J *Rag1^-/-^* mice. The less severe pathology could be due to differences in duration of infection (1 month in the current study *vs* 3–5 months in the earlier study), the different mouse strains, and/or the effect of pregnancy on immune responses and development of inflammation. Additional studies directly comparing *B. miyamotoi* infection in BALB/C and C57BL/6J *Rag1^-/-^* mice under the same experimental conditions would be necessary to confirm these differences. We also did not observe pathology or inflammation in the uteri, the yolk sacs or placenta of pregnant dams. Spirochetes were plentiful in all those tissues, in particular in the maternal blood sinuses between the uterus and placenta, but no spirochetes were observed in embryonic pups (**Figure 7**). Our results demonstrating placental infection are consistent with a report assessing the presence of *Borrelia* species in Canadian wildlife, in which *B. miyamotoi* was found in the placentae of two late-stage fetuses from a pregnant, jumping mouse (*Napaeozapus insignis*) co-infected with *B. burgdorferi* and *B. miyamotoi* (64).

Maternal anemia alone has been found to cause decreased pup survival in mice with both reduced numbers of live births and survival of neonatal pups to weaning (65–68). We observed anemia with *B. miyamotoi* infections of *Rag1^-/-^* mice that became more severe during pregnancy. Anemia was also observed in uninfected pregnant dams at embryonic gestation day 18.5, although less severe than in infected pregnant dams. It is possible that anemia alone could lead to the reduced pup survival rates in the *Rag1*^-/-^ mice independent of infection status. Additional studies are warranted to determine if the anemia that results from infection is the main driver of the decreased pup survival in the *Rag1^-/-^* mice.

Louse and soft tick RF spirochetes are known to lead to adverse outcomes of human pregnancies complicated by these infections (13, 69, 70). In particular, *B. duttonii*, a soft tick RF found predominantly in sub-Saharan Africa causes significant morbidity and mortality to an unborn fetus (18, 71). A WT C3H/HeN mouse model of *B. duttonii* infection during pregnancy documented features similar to those observed with infection with this pathogen during human pregnancy, including intrauterine growth retardation, placental damage and inflammation, impaired fetal circulation, and decreased maternal hemoglobin levels (49). Spirochetes frequently crossed the maternal-fetal barrier, leading to infection of the fetus (49). Anemia has been observed during soft tick RF infections (72), although it is more severe in non-pregnant *B. duttonii* infected mice than in pregnant mice harboring this pathogen. While we observed similar hematologic abnormalities and reduced fetal survival in CT14D4-infected *Rag1^-/-^* mice, notably transplacental transmission of spirochetes to embryos was not found. In contrast to *B. duttonii* infection, infection of WT mice with CT14D4 did not appear to have adverse effects on pregnancy outcome.

Little is known about the impact of hard tick RF infections on pregnancy outcomes in humans. Currently, two case reports have been published describing *B. miyamotoi* infection during the late 2^nd^- early 3^rd^ trimester of pregnancy (27 weeks and 33 weeks). Both individuals experienced severe illness with leukopenia, thrombocytopenia and anemia requiring hospitalization (37, 38). In each case, broad-spectrum antibiotics as well as antiviral agents were administered prior to laboratory testing supporting *B. miyamotoi* infection. Both people improved clinically and no adverse effects on their fetuses were observed, with one report documenting vaginal delivery of a healthy neonate with normal weight at 41 weeks of gestation (38). In our studies, neither *Rag1*^-/-^ nor WT dams exhibited the severe illnesses after CT14D4 infection documented in the human cases, although lymphopenia and anemia were observed in the *Rag1*^-/-^ mice. Large-scale prospective studies of pregnant people in areas highly endemic for *B. miyamotoi* would be required to better understand the risk of *B. miyamotoi* during pregnancy and also the applicability of mouse studies to human infection during pregnancy with this pathogen.

The conclusions from our studies are limited in part by the varied factors that can affect the success of breeding, pregnancy and pup survival in laboratory animal facilities. Due to the number of mice needed to conduct these pregnancy studies comparing outcomes in infected and uninfected mice, we were unable to compare immunodeficient and WT mice in the same experiment. In addition, the control group numbers were often small which may limit some statistical analyses or the identification of statistical differences. We conducted only a single exploratory experiment in WT mice infected prior to breeding that documented a small proportion of pups not surviving after birth until weaning. However, litters born to those same dams bred a second time after they had cleared infection had significantly higher (100%) pup survival rates. The dams were 1–2 months older for the second breeding and more experienced mothers, which could account for these findings. It is also possible that tick transmitted infection of WT mice could result in more adverse consequences. While the BALB/c background is not considered to have high fecundity and pregnancy outcomes (https://www.informatics.jax.org/silver/chapters/4-1.shtml), we chose this mouse strain as in our animal facility, pregnant BALB/C *Rag1^-/-^* dams often have larger litter sizes and are better at successfully raising pups to weaning compared to other commonly used *Rag1^-/-^* laboratory mouse strains (Carmen Jane Booth, personal communication). Conducting most of our studies in *Rag1-/-* mice limited our ability to assess the role of T and B lymphocytes in pregnancy outcomes. Finally, even though mice and humans have hemochorial placentae, there are anatomical differences between placentae of the two species that could influence the susceptibility for maternal to fetal transmission of *B. miyamotoi* infection in humans. Human placentae have only two trophoblast layers that separate maternal and fetal blood that is reduced to a single layer later in gestation, whereas mice have 3 cell layers that are maintained throughout pregnancy (59, 62, 73). Therefore, it is possible that in humans *B. miyamotoi* spirochetes could more easily be transmitted from the placenta to infect the fetus.

In summary, we demonstrate that *B. miyamotoi* of immunodeficient *Rag1-/-* can have negative impacts on pregnancy, including loss of embryonic pups *in utero*, neonatal pup death, and potential perinatal infection of pups. However, we found no evidence of embryonic infection or inflammation in the placentae making it unlikely that the negative impacts of *B. miyamotoi* infection on pregnancy outcomes are due to inflammatory responses to the spirochetes. Given our observations in immunodeficient mice, future studies to more systematically address potential complications of *B. miyamotoi* infection in WT mice are warranted. Our experiments in the mouse model and the case reports of *B. miyamoto*i infection during human pregnancy also emphasize the need to consider this infection in pregnant women presenting with high fever and possible tick-borne illnesses in regions where *B. miyamotoi* is endemic, as prompt institution of appropriate antibiotics may reduce adverse outcomes (74, 75).

## Data Availability

The original contributions presented in the study are included in the article/**Supplementary Material**. Further inquiries can be directed to the corresponding author.
