# Supplementary material for: Borrelia miyamotoi infection negatively impacts pregnancy outcomes in immunodeficient mice
Source: Front Immunol. 2026 Jul 6;17:1870226. doi: 10.3389/fimmu.2026.1870226 (PMC13381220; doi:10.3389/fimmu.2026.1870226)
Supplement: Supplementary file 1 [file DataSheet1.pdf]

Supplemental figure 1

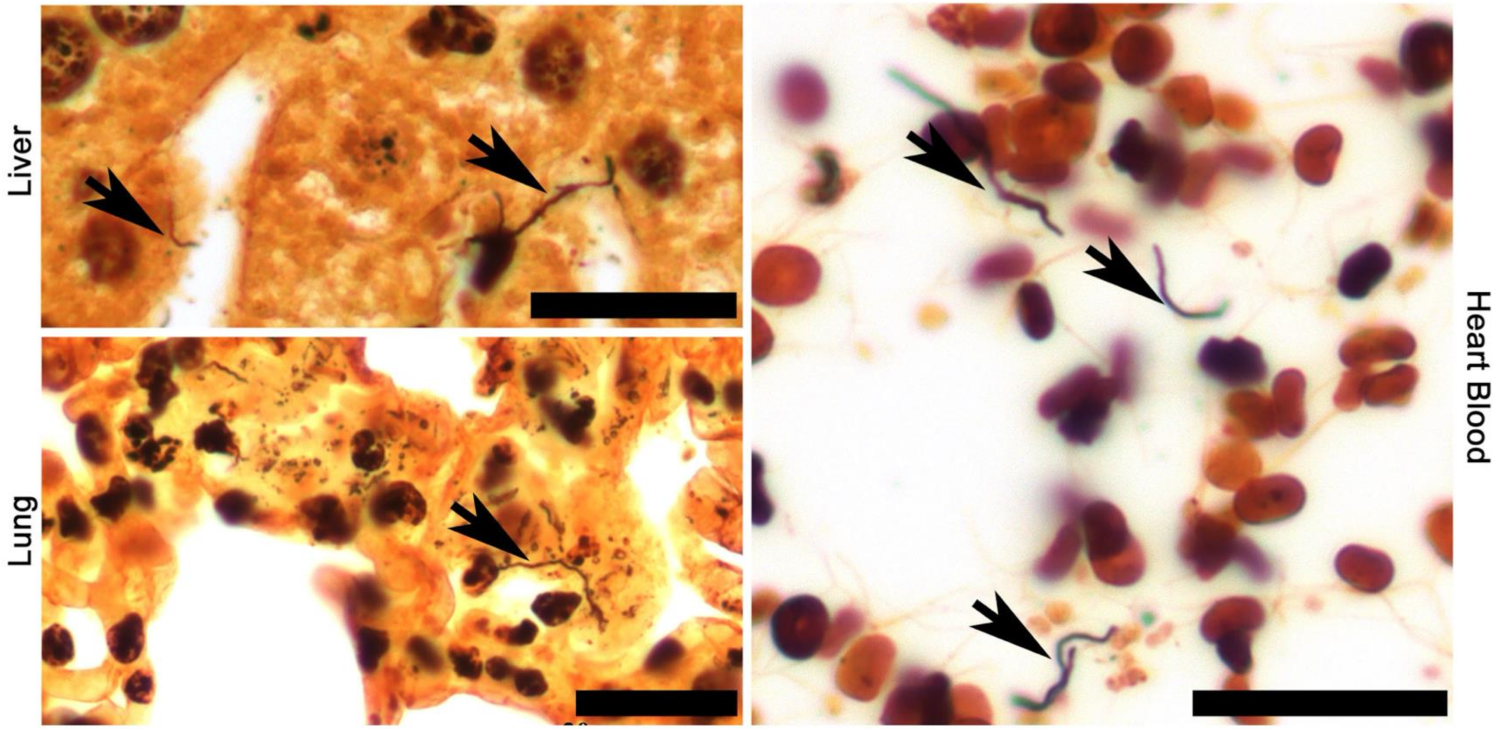

Scale Bars = 20µm

Supplemental Figure 1
Warthin-Starry (WS) stained tissues from tick-infected dams taken at embryonic gestation day 18.5 (see figure 3) show evidence of spirochetes in liver, lung, and blood within the heart tissue which serve as positive controls for identification of spirochetes in all tissues examined (spirochetes indicated by arrows in each section). The mice were not perfused, so blood still remains in all of the tissues. One representative stained section from each tissue type is shown as all tissue sections from each of the 7 infected dams had spirochetes.

Supplemental figure 2

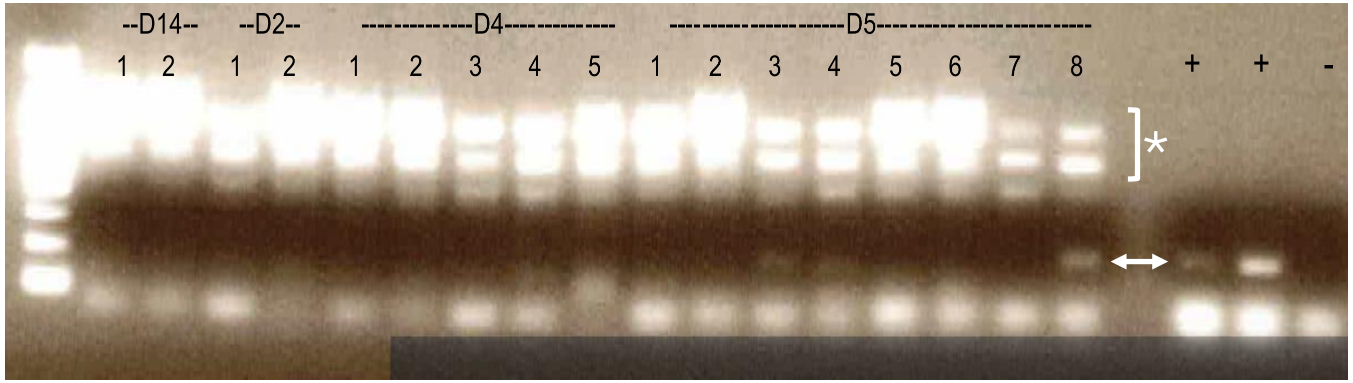

Supplemental Figure 2.
PCR analysis for *B. miyamotoi flaB* gene (see methods) from blood of WT weaned mice (See figure 9) show evidence of *B. miyamotoi* DNA in a small subset of the pups. The double-headed white arrow shows the size of the *flaB gene* PCR product in the pups (left of arrow) and positive control *B. miyamotoi* DNA (right of arrow). The white bracket with asterisk denotes non-specific amplicons from the PCR reaction. DNA from subsets of pups (each litter is separately numbered) from 4 litters are shown, and the litters from each dam are designated as D14, D2, D4 and D5.
