# Supplementary material for: Borrelia miyamotoi infection negatively impacts pregnancy outcomes in immunodeficient mice
Source: Front Immunol. 2026 Jul 6;17:1870226. doi: 10.3389/fimmu.2026.1870226 (PMC13381220; doi:10.3389/fimmu.2026.1870226)
Supplement: Supplementary file 2 [file Table1.pdf]

Supplemental Table 1. Summary of data and assays completed in the 4 Experiments described in the *Results*.

	Expt 1		Expt 2		Expt 3			Expt 4	
Mice BALB/C	*Rag 1-/-*		*Rag 1-/-*		*Rag 1-/-*			WT	
Age	9-13 week		8-10 weeks		12-18 weeks		16-17 weeks	9-11 weeks	15-17 weeks
Intervention	Infected 9 days after breed	Controls none	Infected 7 days prior to breed	Controls none	Infected Tick infested 9 days prior to breed (3 unbred)	Uninfected Tick infested 9 days prior to breed (2 unbred)	Infected Tick infested 9 days prior to breed	Infected 7 days prior to breed	Infected 51 days prior to breed
Mode of infection	needle		needle		tick		tick	needle	
Housing after breeding	Plug + females singly housed		Female & male co-housed until birth		Plug + females singly housed		Plug + females singly housed	Plug + females singly housed	Plug + females singly housed
Numbers of females bred	22		10	10	22	11	4	20	11
Number of pregnant dams	5	3	10	10	10	5	3	17	9
Dams with live births	5	3	6	10	3	2		17	9
Litter size range	1-5	3-6	4-10	3-11	3-5	5-6		2-10	2-11
Live pup births	16	15	34	66	13	11		100	51
Pups surviving to weaning	3	10	24	52	2	9		87	51
Pups with infection at weaning	0		7		1			0	0
Dams sacrificed at d18.5					7	3	3		
D18.5 litter size					2-13	5-9	3 per dam used for analyses		
D18.5 embryos					61	22			
Warthin-Starry staining					assessed	assessed	assessed		
H&E staining					assessed	assessed	assessed		
CD68 staining							assessed		
CBC					assessed	assessed			

The Experiment numbers in the table correspond to Figures 1-4 depicting the different experimental designs. Gray boxes indicate that data/samples were not collected for those measurements.
